# Thickness Asymmetry of the Vertebral Artery Groove: A Dried Vertebrae Study

**DOI:** 10.7759/cureus.58206

**Published:** 2024-04-13

**Authors:** Christos Lyrtzis, Athina Stamati, Parmenion P Tsitsopoulos, Maria Piagkou, Konstantinos Natsis

**Affiliations:** 1 Department of Anatomy and Surgical Anatomy, Aristotle University of Thessaloniki, Thessaloniki, GRC; 2 Department of Neurosurgery, Hippokration General Hospital, Aristotle University of Thessaloniki, Thessaloniki, GRC

**Keywords:** morphometry, study, asymmetry, atlas, vertebral artery groove

## Abstract

Background: The vertebral artery groove (VAG), located on the posterior arch of the first cervical (atlas) vertebra plays a pivotal role in guiding the vertebral artery’s (VA) third part (V3). Deviations in VAG morphology and morphometry (dimensions) can influence vascular dynamics and pose clinical implications.

Aim: The current study delves into the morphometric variants and explores the less-explored morphometric variable of the VAG thickness, highlighting possible laterality (asymmetry).

Methods: A morphometric investigation was conducted on 141 dried atlas (73 male and 68 female) vertebrae from a Greek adult population. The VAG's minimum thickness was investigated by considering the laterality (sides’ differences), gender, and age impact on it. Measurements were performed by two independent researchers, ensuring the data reliability.

Results: A significant asymmetry was identified in the VAG thickness between the left (3.9 ± 0.9 mm) and right (4.1 ± 1.1 mm) (p=0.005) sides, with the left side having the mean minimum thickness. Gender had a significant impact on VAG thickness only on the left side, with females presenting a significantly thinner left-sided VAG (3.6 ± 0.9 mm) than males (4.10 ± 0.7 mm) (p=0.001). Age had no significant impact on the VAG thickness.

Conclusion: The present study underscores the significance of asymmetry in the VAG thickness in craniocervical interventions. This less-explored morphometric variable warrants careful consideration by surgeons during preoperative planning to minimize potential complications. The current findings highlight the importance of understanding the VAG thickness asymmetry and its clinical implications, as this osseous variable may be an index of a different diameter of the VA by side. It is recommended that surgeons incorporate this variable into their preoperative assessments to improve the safety and efficacy of craniocervical interventions.

## Introduction

A comprehensive understanding of the musculoskeletal and neurovascular anatomy of the upper cervical spine is imperative during surgery to prevent severe vascular injury [1]. Advances in imaging and surgical techniques have significantly improved the early diagnosis and treatment of spinal disease and injury, particularly in the complex craniocervical (occiput-atlas and axis vertebrae) junction [2]. To achieve successful anterior and posterior interventions of the atlas (first cervical) vertebra area and minimize the risk of complications, surgeons must possess a deep understanding of the atlas variants, biomechanics, and adjacent vertebral artery (VA) morphology.

Detailed examination of the atlas vertebra features is clinically crucial, as several instrumentations (wiring, trans-articular atlantoaxial screw fixation, and the use of hooks and screws) are inserted into the atlas lateral masses, following various interventions [3]. VA iatrogenic injury is a severe perioperative complication with potentially devastating consequences [4,5]. The vertebrobasilar arterial system, also known as the posterior circulation, is the second-largest blood supplier to the brain [6]. The VA’s suboccipital segment, constituting its third part and symbolized as V3, courses over the atlas posterior arch upper surface, posterior to the lateral mass, forming an impression known as the VA groove (VAG) [7-11]. From the morphological point of view, the partial or complete ossification at the upper part of the atlas posterior arch may result in an incomplete or complete posterior ponticulous (bridge). This complete bridge results in an arcuate foramen (AF) [12,13] which may compress the V3, leading lead to ischemia [14].

Studies have emphasized the significance of VAG morphology in surgical procedures [15]. Gupta's morphometric analysis of the atlas vertebrae highlighted the importance of safe distances during V3 mobilization, providing critical information for surgical planning [16]. Rocha et al. emphasized the importance of working area and safety zones during screw placement on the atlas lateral mass, reinforcing the surgical necessity for precise anatomical knowledge, targeting surgical outcomes’ optimization and complications’ minimization [17]. The current dried atlas vertebrae study documents the VAG minimum thickness, identifies potential side-related differences, and explores the gender and age impact.

## Materials and methods

The investigation was conducted on dried atlas vertebrae obtained from a Greek adult sample. The specimens used were sourced from a well-documented collection of the Department of Anatomy and Surgical Anatomy of the Aristotle University of Thessaloniki, Greece, supplemented by contributions from cemeteries in Thessaloniki and Serres regions. The Ethics Committee of the Aristotle University of Thessaloniki approved the current study’s protocol (as part of a thesis protocol, PN 5343/12.12.2018). The Helsinki Declaration (1964), a set of ethical principles regarding human tissues and research, was taken into consideration. This study has been exempted from the need for approval by the Institutional Review Board (IRB). This is because the samples, namely the bones, used in the study already belong to a collection available for educational or research purposes by the anatomy lab.

**Figure 1:**
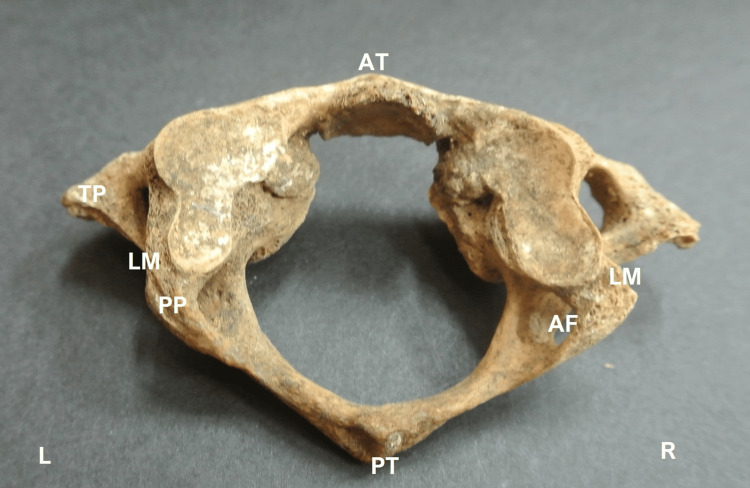
The arcuate foramen (AF) after the complete ossification of the ligament posterior to the lateral mass (LM), thus creating a posterior ponticulous (PP). TP: transverse process; PT: posterior tubercle of the atlas; AT: anterior tubercle; R: right; L: left

**Figure 2:**
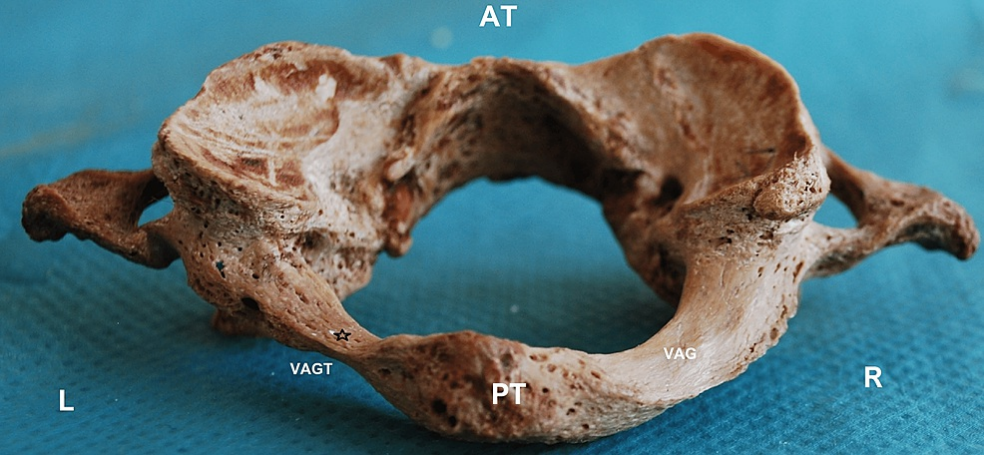
The calculation of the vertebral artery groove minimum thickness (VAGT) depicted by the black asterisk at the left side of the VAG. L: left; R: right side; AT; anterior tubercle; PT: posterior tubercle

A total of 141 dried atlas vertebrae were finally collected from 73 male and 68 female Greek adult individuals. The vertebrae were further classified into three age groups (Group 1 (20-39 years of age; 19 vertebrae), Group 2 (40-59 years; 36 vertebrae), and Group 3 (60-79 years; 86 vertebrae)) to examine the age impact on VAG minimum thickness. To ensure the measurements’ reliability and objectivity, two independent and qualified anatomists performed the assessments. The calculation of the VAG minimum thickness at the C1 posterior arch was performed in mm (Figures 1, 2), using a digital sliding caliper (Mitutoyo ABSOLUTE 500-196-20 model; Mitutoyo Corporation, Kanagawa, Japan), with an accuracy of 0.01 mm. The inclusion of demographic information, the blinded measurement approach, and the statistical rigor enhanced the research's scientific validity, facilitating meaningful comparisons with other populations. All vertebrae were free of malformations, disease, previous trauma, and surgery. Broken vertebrae were excluded from the study. The length of the VAG was not included in the study, as the diversity of methodologies used among published studies made intercomparisons impossible.

Statistical analysis

Statistical analysis was carried out using IBM SPSS Statistics for Windows, Version 22.0 (Released 2013; IBM Corp., Armonk, New York, United States), ensuring the data robust processing. The VAG minimum thickness was compared by using the independent samples t-test. To assess the asymmetry between the two sides (laterality), the paired samples t-test was utilized. For examining the gender and age impact on the VAG minimum thickness, the Mann-Whitney U-test and ANOVA test were applied.

## Results

The minimum VAG thickness was significantly lower for the left side (3.86 ± 0.87 mm) compared to the right side (4.13 ± 1.11 mm) (Table 1). The VAG thickness showed asymmetry (p=0.005) (Table 2). The VAG thickness was significantly smaller (P=0.001) in females (3.59 ± 0.95 mm) compared to males (4.11 ± 0.71 mm) only on the left side (Table 1). No significant age effect on the VAG thickness was observed (p > 0.05) (Table 2).

**Table 1:** Minimum vertebral artery groove (VAG) thickness on the right and left side, expressed in mm.

	VAG thickness (mm)			
	Right side			
Total, mean ± SD (range)	4.1 ± 1.1 (2.01-7.68)			
Gender	Male		Female	
Mean ± SD (range)	4.3 ± 1.1 (2.29-7.45)		4.0 ± 1.1 (2.01-7.68)	
p-value	0.135 (no impact to side), Mann Whitney U Test			
Age groups	20-39 years	40-59 years		60-79 years
Mean ± SD (range)	3.9 ± 1.1 (2.01-6.51)	3.95 ± 0.9 (2.04-6.32)		4.2 ± 1.2 (2.52-7.68)
p-value	0.528 (no impact to side), ANOVA			
	Left side			
Total, mean ± SD (range)	3.9 ± 0.9 (2.0-8.2)			
Gender	Male		Female	
Mean ± SD (range)	4.1± 0.7 (2.2-5.7)		3.6 ± 1.0 (2.0-8.2)	
p-value	0.001 (significant difference), Mann Whitney U Test			
Age groups	20-39 years	40-59 years		60-79 years
Mean ± SD (range)	3.8 ± 0.8 (2.0-5.0)	3.8 ± 0.8 (2.0-5.6)		3.9 ± 0.9 (2.0-8.2)
p-value	0.846 (no impact to side), ANOVA			

**Table 2:** Comparison of the minimum thickness on the right and left vertebral artery groove (VAG) and the expressed asymmetry.

Minimum thickness of the VAG (mm)	
Right Side, mean ± SD	Left Side, mean ± SD
4.1 ± 1.11 (thicker)	3.9 ± 0.9
p-value	
0.005 (asymmetry)	

## Discussion

In the current morphometric dried atlas vertebrae study concerning the VAG minimum thickness, we provide critical insights for spine surgeons engaging in craniovertebral junctions by using several approaches. Asymmetry in the VAG minimum thickness can be attributed to developmental and genetic factors, leading to atlas variant morphology [13,18]. A fusion of costal elements of the atlas may explain the formation of abnormal morphological variants, such as VAG asymmetry, accessory grooves, or foramina [13,19]. This asymmetry may have implications in fields such as forensic science and surgery where population-specific differences, including gender information, need to be considered [1,20].

VAG thickness

The current study's VAG minimum thickness was 4.1 ± 1.1 mm on the right and 3.9 ± 0.9 mm on the left side, with an evident asymmetry. Gender-significant differences were observed with female vertebrae presenting a lower thickness (3.6 ± 1.0 mm) compared to male (4.1 ± 0.7 mm, p=0.001), on the left side. On the right side, higher mean values were identified on both genders, with female vertebrae having less thickness (4.0 ± 1.1 mm), than male (4.3 ± 1.1 mm). No age impact was identified. Comparisons with other studies conducted in several populations highlight VAG variant thickness [14,21,22], expressing a possible asymmetry in V3 diameter bilaterally, with the left V3 often larger than the right one adding complexity to the VAG morphology understanding [23,24] (Table 3). Similar mean values to the current study were identified in another study conducted on a Greek population by Natsis et al. [13] and in studies conducted in India and United States geographic regions by Patel and Gupta [25] and Ebraheim et al. [9]. The minimum mean VAG thickness was recorded by Rekha and Divya Shanthi [26] in an Indian population study (Table 3).

**Table 3:** Vertebral artery groove (VAG) thickness in the literature according to the observed side (right and left)

Authors	Year	Thickness of VAG (mm)	
		Right side, mean ± SD	Left side, mean ± SD
Ebraheim et al. [9]	1998	4.1 ± 1.2 (total)	
Tan et al. [22]	2003	4.72 ± 0.68	4.58 ± 0.65
de Carvalho et al. [4]	2009	3.87 ± 0.83	3.92 ± 1.10
Awadalla and Fetouh [3]	2009	4.48 ± 0.9	4.49 ± 0.9
Ravichandran et al. [21]	2011	4.7 ± 0.98	4.55 ± 0.84
Gosavi and Vatsalaswamy [27]	2012	3.72 ±1.06	3.70 ±1.06
Ansari [7]	2015	3.79 ±1.08	4.05±0.086
Patel and Gupta [25]	2016	4.15 ±1.28	3.99±1.28
Rekha and Divya Shanthi [26]	2016	3.68	3.70
Natsis et al. [13]	2019	4.13 ± 1.11	3.86 ± 0.87
Current Study	2024	4.1 ± 1.1	3.9 ± 0.9

Study limitations 

The study primarily focuses on a specific group of Greek adults, which may limit the applicability of the findings to other populations. The use of cadaveric specimens, while beneficial in understanding anatomy and adding further useful details concerning the relationships to VA diameters, may also introduce limitations due to the preservation state and potential variants in the sample population. Additionally, the measurements’ accuracy may be impacted by post-mortem changes and individual differences. The study's cross-sectional design also prevents exploration of dynamic factors that may influence the VAG thickness over time. Therefore, longitudinal studies are necessary to capture age-related changes and enhance our understanding of structural alterations.

Despite these limitations, the present dried atlas vertebrae study lays the groundwork for exploring the VAG minimum thickness by side, gender, and age, which can be a valuable clinical reference. Future larger investigations that incorporate diverse populations, advanced imaging techniques and spinal instrumentation, and longitudinal assessments can refine the current knowledge and contribute to more comprehensive insights into the craniovertebral junction.

## Conclusions

Side difference was noted in the VAG thickness, with the right side being thicker than the left, and a gender dimorphism expressed in this minimum thickness. The current findings highlight the importance of understanding the VAG thickness asymmetry and its clinical implications, as this osseous variable may be an index of a different diameter of the VA by side. It is recommended that surgeons incorporate this variable into their preoperative assessments to improve the safety and efficacy of craniocervical interventions. Moreover, it is crucial to take into consideration possible differences in the VAG morphometrical variability (asymmetry) in different populations. Surgeons must consider population, gender, and age variants, as well as any existing anatomical-morphological differences, to make informed decisions regarding surgical access and guiding points. This is vital in avoiding complications that may arise from potential injury to the VA, and adjacent neural elements.
